# Follow-up after treatment for head and neck cancer: United Kingdom National Multidisciplinary Guidelines

**DOI:** 10.1017/S0022215116000645

**Published:** 2016-05

**Authors:** R Simo, J Homer, P Clarke, K Mackenzie, V Paleri, P Pracy, N Roland

**Affiliations:** 1 Department of Otolaryngology – Head and Neck Surgery, Guy's and St Thomas’ Hospital NHS Foundation Trust, Guy's, King's and St Thomas’ Medical and Dental School, London, UK; 2 Department of Otolaryngology – Head and Neck Surgery, Manchester Royal Infirmary and Christie Hospital, University of Manchester, Manchester, UK; 3 Department of ENT, Charing Cross and Royal Marsden Hospitals, London, UK; 4 Glasgow Royal Infirmary, University of Glasgow, Glasgow, UK; 6 Department of ENT Head and Neck Surgery, Queen Elizabeth Hospital, Birmingham, UK; 5 Department of Otolaryngology-Head and Neck Surgery, The Newcastle upon Tyne Hospitals NHS Foundation Trust, Northern Institute of Cancer Research, Newcastle upon Tyne, UK; 7 Department of Otolaryngology-Head and Neck Surgery, Aintree University Hospitals NHS Foundation Trust, Liverpool, UK

## Abstract

This is the official guideline endorsed by the specialty associations involved in the care of head and neck cancer patients in the UK. In the absence of high-level evidence base for follow-up practices, the duration and frequency are often at the discretion of local centres. By reviewing the existing literature and collating experience from varying practices across the UK, this paper provides recommendations on the work up and management of lateral skull base cancer based on the existing evidence base for this rare condition.

**Recommendations:**

• Patients should be followed up to a minimum of five years with a prolonged follow-up for selected patients. (G)

• Patients should be followed up at least two monthly in the first two years and three to six monthly in the subsequent years. (G)

• Patients should be seen in dedicated multidisciplinary head and neck oncology clinics. (G)

• Patients should be followed up by dedicated multidisciplinary clinical teams. (G)

• The multidisciplinary follow-up team should include clinical nurse specialists, speech and language therapists, dietitians and other allied health professionals in the role of key workers. (G)

• Clinical assessment should include adequate clinical examination including fibre-optic rigid or flexible nasopharyngolaryngoscopy. (R)

• Magnetic resonance imaging and positron emission tomography combined with computed tomography imaging should be used when recurrence is suspected. (R)

• Narrow band imaging can be used in the follow-up in selected sites. (R)

• Second primary tumours should be part of rationale of follow-up and therefore adequate screening strategies should be used to detect them. (G)

• Patients should be educated with regard to the appearance and detection of recurrences. (G)

• Patients with persistent pain should be investigated to exclude recurrent disease. (R)

• Patients should be offered support with tobacco and alcohol cessation services. (R)

## Introduction

It is accepted that the follow-up of patients who had treatment for head and neck cancers is a fundamental part of their care.^1^^–^^4^ The reasons of post-treatment follow-up include:

• Evaluation of treatment response
• Early identification of recurrence
• Early detection of new primary tumours
• Monitoring and management of complications
• Optimisation of rehabilitation
• Provision of support to patients and their families.

Controversy exists in how these aims are achieved.^5^^,^^6^ Increasing efforts are being made to rationalise the structure and timing of head and neck follow-up clinics.

The general structure of follow-up clinics is to have initial high-frequency visits especially in the first two years when the risk of loco-regional recurrence is known to be high and then reduce frequency, with follow-up often finishing at five years. In the UK, the structure of these clinics is often arbitrary and reflects institutional and clinician-led practices with very little evidence to support any one system.

Evidence to support follow-up for early detection of tumour recurrence is lacking. However, there is a belief that follow-up clinics have inherent value and to date all published studies recognise this fact.^7^

In order to rationalise follow-up, patients could be divided into low and high risk. This is well recognised in thyroid cancer, but it is not the case in all other types of head and neck cancer especially squamous cell carcinoma (SCC). It is a belief that, this categorisation could help to determine which patients should be followed for more than five years. It would also help to establish which screening test may be needed in order to detect recurrence or second primaries.

## General considerations

### Length

The length of follow-up is generally five years although there are many clinicians who follow-up patients for longer periods or even for life.^8^ Follow-up of patients over five years would be justified for the following groups: high-risk patients, specific tumours (e.g. adenoid cystic carcinomas), patients who have undergone complex treatments who require on-going rehabilitation and support, detection of new primary tumours and patient preference. Fear of recurrence is prevalent in cancer patients and continued attendance at clinic helps to mitigate this.

**Recommendation**

• Patients should be followed up to a minimum of five years with a prolonged follow-up for selected patients (G)

### Frequency

At present, there is no evidence that high frequency of follow-up visits confers any benefit in terms of morbidity and mortality. However, there is evidence that the majority of clinicians in the UK support the follow-up of patients, in regular high-frequency intervals in the first two years when the risk of locoregional recurrence is high followed by a decrease in frequency after the second year. The follow-up in the first two years should be between four to eight weeks and from three to six months thereafter.^7^

**Recommendation**

• Patients should be followed up at least two monthly in the first two years and three to six monthly in the subsequent years (G)

### Setting

At present, 90 per cent of the clinicians treating head and neck cancer in the UK see the patients in dedicated head and neck clinics for the duration of the follow-up.

**Recommendation**

• Patients should be seen in dedicated multidisciplinary head and neck oncology clinics (G)

### Type of health professional

At present patients are followed up by their treating clinicians and their teams. Allied health professionals including speech and language therapists, dieticians and clinical nurse specialists may offer specific follow-up in their areas of expertise, but this is usually in addition to the medical follow-up. The introduction of the clinical nurse specialist and the key worker role in the management of patients with head and neck cancer has opened lines of communication between the patient and family and the clinical team^9^ should any problems arise.

**Recommendations**

• Patients should be followed up by the dedicated multidisciplinary clinical teams (G)
• The multidisciplinary follow-up team should include clinical nurse specialists, speech and language therapists, dietitians and other allied health professionals in the role of key workers (G)

### Clinical assessment

Traditionally, clinical assessment has been the most important aspect of the follow-up in patients treated for head and neck cancer. The clinical evaluation is done by inspection, palpation and at present with fibre-optic rigid or flexible nasopharyngolaryngoscopy. Rigid stroboscopy can also be used in patients who have been treated for laryngeal cancer.

**Recommendation**

• Clinical assessment should include adequate clinical examination, including fibre-optic rigid or flexible nasopharyngolaryngoscopy (R)

### Screening investigations

Currently there is evidence that magnetic resonance imaging (MRI) and positron emission tomography combined with computed tomography (PET–CT) scanning are superior at detecting recurrence and second primaries.^10^^,^^11^ This is especially true in some tumour sites such as the nasopharynx and following treatment with chemo-radiation. Positron emission tomography combined with computed tomography has also the advantage of being a systemic evaluation. Diffusion-weighted MR has been recently applied with promising results; however, its accurate interpretation requires specific training and experience. Narrow band imaging^12^ (NBI), possibly associated with high definition television technology, has been shown to be an adjunctive imaging tool due to its specific capability to selectively address superficial persistences and/or recurrences or second primary tumours by enhancing their pathognomonic neoangiogenetic pattern. It has been reported that its use can detect 18 per cent more true positive laryngeal cancerous lesions than conventional white light endoscopy. This is true even after radiotherapy (RT) or chemoradiotherapy, due to the high accuracy (98 per cent) of NBI in differentiating between neoplastic disease and post-RT inflammatory and/or cicatricial changes.

**Recommendations**

• Magnetic resonance imaging and PET–CT imaging should be used when recurrence is suspected (R)
• Narrow band imaging can be used in the follow-up in selected sites (R)

### Second primary tumours

The incidence of second primary tumours varies between 5 and 12 per cent at five years. There is good evidence to indicate that patients with head and neck SCC have an increased risk of developing second primary malignant tumours.^13^^,^^14^ This risk appears to be constant throughout the follow-up period, with an incidence ranging from 2 to 4 per cent per year. Traditionally, patients undergoing follow-up for head and neck cancer underwent a chest radiograph every year. However, there is evidence that these have not been able to identify metastasis with any confidence.

**Recommendation**

• Second primary tumours should be part of rationale of follow-up and therefore adequate screening strategies should be used to detect them (G)

## Specific considerations

### Second-look microlaryngoscopy

In laryngeal cancer, especially in those patients treated with transoral laser microsurgical excision, it is advisable to perform second-look microlaryngoscopy,^15^ especially in scenarios where there is lack of agreement between the intraoperative and histological findings regarding the completeness of resection. The rationale of this is to provide evidence of complete resection, detect residual tumour and to perform further treatment should this be necessary.

### Patients with persistent or recurrent pain without clinical evidence of disease

Pain complaints must be regarded as a serious warning sign of recurrent disease during follow-up of HNC patients,^16^^,^^17^ even in the absence of an endoscopically visible persistence and/or recurrence. Persistent neck pain can be the first symptom of recurrent disease in 70 per cent of patients and can be an independent predictor of both recurrence and five-year survival rate. Pain should always prompt the clinician to initiate a thorough set of investigations, both by imaging and/or endoscopy under general anaesthesia, in order to reduce possible diagnostic delays. Pain without endoscopic evidence of disease is more frequently encountered after RT or chemoradiotherapy, but it is possible even after surgery. This symptom is usually caused by submucosal disease recurrence possibly hidden by oedematous mucosa, or associated with chondritis, chondronecrosis or osteonecrosis as a result of previous treatments.

### Tumour markers

There is no evidence that the use of tumour markers is any value in the follow-up of patients with head and neck SCCs. The use of tumour markers in the follow-up of patients with thyroid cancer is addressed elsewhere in these guidelines.

### Patient education

It has been recognised that the education of patients plays an essential role in the detection of recurrences. The vast majority of recurrences are diagnosed following the occurrence of new symptoms and thus patients should be educated about the need to seek help when appropriate. It has also been recognised that continuing smoking and alcohol drinking increases the risk of recurrence and second primary tumours. It is therefore imperative that patients are advised and offered support with regards to the detrimental effects of tobacco smoking and alcohol addiction.

**Recommendations**

• Patients should be educated with regards to the appearance and detection of recurrences (G)
• Patients with persistent pain should investigate to exclude recurrent disease (R)
• Patients should be offered support with tobacco and alcohol cessation services (R)

### Key points

• The aims of follow up of patients after treatment for head and neck cancers are manifold
• The frequency of follow-up is higher in the first two years, with reduced frequency subsequently, finishing at five years
• Medical, nursing and allied health professionals all play important roles in providing follow up care
• Change in patient symptoms during follow up is the most frequent indication of recurrent disease and must be regarded seriously, even if clinical examination reveals no abnormalities.
